# The Effects of Early Post-Operative Soluble Dietary Fiber Enteral Nutrition for Colon Cancer

**DOI:** 10.3390/nu8090584

**Published:** 2016-09-21

**Authors:** Rui Xu, Zhi Ding, Ping Zhao, Lingchao Tang, Xiaoli Tang, Shuomeng Xiao

**Affiliations:** Department of Gastrointestinal Surgery, Sichuan Cancer Hospital, Chengdu 610041, China; xrscu2012@163.com (R.X.); dzscch@sina.com (Z.D.); zpscch@sina.com (P.Z.); scch2015@163.com (L.T.); txlscch@sina.com (X.T.)

**Keywords:** colon cancer, dietary fiber, early enteral nutrition

## Abstract

We examined colon cancer patients who received soluble dietary fiber enteral nutrition (SDFEN) to evaluate the feasibility and potential benefit of early SDFEN compared to EN. Sixty patients who were confirmed as having colon cancer with histologically and accepted radical resection of colon cancer were randomized into an SDFEN group and an EN group. The postoperative complications, length of hospital stay (LOH), days for first fecal passage, and the difference in nutritional status, immune function and inflammatory reaction between pre-operation and post-operation were all recorded. The statistical analyses were performed using the *t*-test and the chi square test. Statistical significance was defined as *p* < 0.05. After the nutrition support, differences in the levels of albumin, prealbumin and transferrin in each group were not statistically significant (*p* > 0.05); the levels of CD4+, IgA and IgM in the SDFEN group were higher than that of the EN group at seven days (*p* < 0.05); the levels of TNF-α and IL-6 in the SDFEN group were lower than that of the EN group at seven days (*p* < 0.05); and patients in the SDFEN group had a significantly shorter first flatus time than the EN group (*p* < 0.05). Early post-operative SDFEN used in colon cancer patients was feasible and beneficial in immune function and reducing inflammatory reaction, gastrointestinal function and speeding up the recovery.

## 1. Introduction

In the 21st century, cancer incidence and mortality has not reduced; moreover, it has even increased year by year. The total number of patients with colorectal cancer increased to 1.23 million globally in 2008; it is ranked third in cancer incidence and fourth in mortality [1]. However, the incidence and mortality of colorectal cancer were ranked third and fifth, respectively [2] in China. Many studies showed that the incidence of malnutrition in patients with a malignant tumor was as high as 31%–97% [3,4], especially for gastrointestinal cancer, so choosing a proper nutrition therapy for colorectal cancer patients with malnutrition is necessary. Early enteral nutrition support is promoted after gastrointestinal surgery, and there are many kinds of nutrient elements, such as probiotics, ω-3PUFA, Gln and fiber. We know that dietary fiber is a kind of nutrient, which is divided into soluble dietary fiber and insoluble dietary fiber and can protect the intestinal barrier, modulate immune function, induce inflammatory response and postoperative complications [5,6]. SCFA is the main source of energy for intestinal epithelial cells and plays a key role in maintaining colonic health and moderating cell growth and differentiation. This depends on the fermentation of dietary fiber. Soluble dietary fiber is fermented, but insoluble dietary fiber is difficult to ferment. The relationship between dietary fiber and colon cancer prevention was studied widely, but there is little study about the early postoperative application of soluble dietary fiber in colon cancer [7,8,9]. Thus, we decide to add soluble dietary fiber to verify the feasibility and potential benefit of SDFEN for colon cancer for the postoperative course.

## 2. Patients and Methods

### 2.1. Patient Selection

We recruited 60 subjects from the department of gastrointestinal surgery of the Sichuan cancer Hospital between June 2014 and June 2015. All patients who confirmed colon cancer with histologically and accepted radical resection of colon cancer were randomized into an SDFEN group (30 cases) and EN group (30 cases). Informed consents were obtained according to the Declaration of Helsinki. Patients suffering from diabetes or underlying cardiopulmonary diseases were excluded. Moreover, the patients whose tumor staging was Stage IV (the cancer had spread to other parts of the body) were also excluded.

### 2.2. Methods

No patients had a decompression tube before the operation, and all were given sequential nutrition support for 7 days consecutively after the operation. The total daily calories for all the patients should reach 125.52 kJ (30 kcal)/kg. All enteral nutrition was ingested through mouth, and if energy was insufficient, we would obtain residual energy by infusing the Fat Emulsion, Amino Acids (17) and Glucose (1%) Injection (1440 mL/bag) made by Sino-Swed Pharmaceutical Corp. (Wuxi, China). Intervention in detail is in the following Table 1.

### 2.3. Clinical Assessment

Clinical factors included age, gender, smoking, drinking habits, and tumor stage according to the tumor-node-metastasis classification of the International Union against Cancer (7th edition) [10]. Peripheral blood was collected on the pre-operative day, and post-operative second and seventh day. Nutritional status was expressed by the levels of albumin, prealbumin and transferring; and immune function was shown by the levels of CD3+, CD4+, CD8+, CD4+/CD8+, IgA, IgM and IgG; The levels of TNF-α, IL-6 and PCT reflected the immune function. Length of hospital stay (LOH), and bowel movement recovery were expressed as days and the first fecal passage was recorded. Postoperative complications including pneumonia, anastomotic fistula and severe abdominal distension were also recorded.

### 2.4. Statistical Analysis

All analyses were carried out with the SPSS 19.0 software package (IBM, New York, NY, USA), and measurement data were indicated with the mean ± standard deviation. The *t*-test was used to examine measurement data, the chi-square test was used to categorize data. *p* value < 0.05 was considered significant for statistical analysis.

## 3. Results

### 3.1. Demography

Among 30 colon cancer cases, there were 16 males and 14 females, and the mean age was 52.20 ± 14.20 years; among the 30 control cases, there were 19 males and 11 females, and the mean age was 53.20 ± 13.10 years. The characteristics of the cases and controls are summarized in Table 2. There was no significant difference between the distributions of the age, gender, smoking, drinking habits and TNM stage of the two groups.

### 3.2. Pre-Operative and Post-Operative Nutritional Index

After the nutrition support, differences of the levels of albumin, prealbumin and transferrin in each group were not statistically significant (*p* > 0.05) (Table 3).

### 3.3. Pre-Operative and Post-Operative Immune Index

Differences of CD3+, CD8+, CD4+/CD8+ and IgG in each group were not statistically significant at two and seven days. The levels of CD4+, IgA and IgM in SDFEN group were higher than the EN group at seven days (*p* < 0.05) (Table 4).

### 3.4. Pre-Operative and Post-Operative Inflammatory Index

The levels of TNF-α and IL-6 in the SDFEN group were lower than the EN group at seven days (*p* < 0.05) (Table 5).

### 3.5. Post-Operative Recovery

Patients in the SDFEN group had a significantly shorter first flatus time than the EN group (*p* < 0.05). However, abdominal distension, pneumonia, Anastomotic fistula and total postoperative hospitalization time were similar between the two groups (Table 6).

## 4. Discussion

Colon cancer is a common gastrointestinal malignancy, the incidence of which has increased year by year. Seventy percent of patients with GI cancer may be suffering from malnutrition and 39.3% of patients with colon cancer had different degrees of malnutrition [11,12]. Surgery is still the first choice for treatment, but surgical trauma and anesthesia can cause body metabolic disorder and aggravated malnutrition. Widespread attention has been given on how to further improve the postoperative nutritional status and the prognosis.

Early enteral nutrition began at 24 h after surgery in our study. ASPEN [13] suggested that ideal enteral nutrition should begin within 24 to 48 h after surgery. Actually, intestine had recovered its absorption function and EMG activity within 4 to 8 h after surgery [14]. The gastrointestinal tract can not only absorb nutrients, but also plays an important role in immunity because it is a central organ after surgical stress [15] and it is also the motor of the MODS. Intestine mucosal atrophy and abnormal intestinal permeability can occur after not eating for several days. Early enteral nutrition is more close to the human physiological needs. The application of enteral nutrition can reverse the loss of gut mucosal integrity resulting from surgical trauma [16], and early enteral nutrition support is associated with a decreased infection risk, a reduced hospital stay, and a clear trend of a reduction in anastomotic breakdown [17,18]. Therefore, it could be the first choice for patients who have had an operation.

Some studies proposed that early application of soluble dietary fiber enteral nutrition could increase nutritional indicators, make patients’ weight decline slowly and reduce the incidence of digestive tract complications [19,20]. However, there was no obvious difference in post-operative nutrition status and complication between the SDFEN group and EN group in our study. Fortunately, there was a trend of an increased post-operative nutrition status and declined incidence of complications. Dietary fiber are not a static collection of indigestible plant materials that pass through the human GI tract without any function; instead, they bind potential nutrients, result in new metabolites, and modulate nutrient absorption/metabolism, and they can promote the growth of the small intestinal villus to increase the absorption of nutrients to improve nutrition status. Dietary fiber is divided into soluble dietary fiber and insoluble dietary fiber. In general, soluble dietary fiber is fermented, but insoluble dietary fiber is difficult to ferment. Studies found that dietary fiber could be fermented into SCFAs in colon and unoxidized SCFA through the portal system into the liver. They can thus be converted to glutamine, and then glutamine enters into the circulation of blood to nourish the small intestine [21]. Therefore, statistical differences in post-operative nutrition status and complication will exist between the SDFEN group and EN group, if observation time is enough.

A large amount of research has reported that soluble dietary fiber had health benefits with immunomodulatory and anti-inflammatory effects [8,22]. In our study, there were significant differences in the indicators of immune conditions including deviations in CD4+, IgA and IgG at seven days between the SDFEN group and EN group (35.90% ± 2.24% vs. 34.41% ± 2.64%, 2.25% ± 0.79 g/L vs. 1.88 ± 0.54 g/L, and 11.55 ± 1.44 g/L vs. 11.41 ± 1.32 g/L, respectively, *p* < 0.05), and the levels of TNF-α and IL-6 in the SDFEN group was lower than those in the EN group (13.02 ± 2.85 pg/mL vs. 14.73 ± 4.07 pg/mL, and 58.75 ± 24.82 pg/mL vs. 70.83 ± 35.65 pg/mL, respectively, *p* < 0.05). These health benefits can be attributed to the fermentation of soluble dietary fiber into SCFAs in the colon. The three major colonic SCFAs are acetate, propionate and butyrate, which is the main source of energy for intestinal epithelial cells and plays a key role in maintaining colonic health and moderating cell growth and differentiation [23]. Acetate plays a role in the host immune system through interacting with the G protein-coupled receptor (GPCR43, 41) in immune cells [24]. Butyrate exhibits strong anti-inflammatory properties, and this effect is likely mediated by inhibition of TNF-α production, NF-κB activation, and IL-8, IL-10, and IL-12 expression in immune and colonic epithelial cells [25,26]. Leukocytes are recruited and migrate from the bloodstream to the inflamed tissue through a multistep process that involves expression and activation of several proteins such as adhesion molecules and chemokines, and SCFAs modify this leukocyte recruitment [27,28] by modulating the amount or type of adhesion molecules and chemokines. SCFAs may alter the recruitment of leukocytes to reduce the chronic GI tract inflammatory response. Therefore, intakes of dietary fiber can reduce inflammatory reaction and improve the postoperative immune function.

Under the stimulation of mixed dietary fiber food, the gastrointestinal hormone is increased, such as gastrin and cholecystokinin, which can promote recovery of intestinal movement. Furthermore, SCFAs also have the same function; Kamath et al. [29] found that the movement of the free small bowel was increased after being stimulated with SCFAs. In our study, patients in the SDFEN group had a significantly shorter first flatus time than the EN group (58.93 ± 6.5 h vs. 63.03 ± 4.8 h, *p* < 0.05), so that SDFEN can promote recovery of intestinal movement.

## 5. Conclusions

Early post-operative soluble dietary fiber enteral nutrition used in colon cancer patients was feasible and has advantages in improving immune function, reducing inflammatory response and promoting early recovery of intestinal movement.

## Figures and Tables

**Table 1:** Intervention of the cases and control.

Time	Nutrients	SDFEN	EN
Day 1	5% GNS	500 mL	500 mL
	dietary fiber	25 g	-
	Probiotics	4g	4 g
Day 2–3	Enteral Nutrition	500 mL	500 mL
	dietary fiber	25 g	-
	Probiotics	4 g	4 g
Day 4–7	Enteral Nutrition	1000 mL	1000 mL
	dietary fiber	25 g	-
	Probiotics	4 g	4 g
Total energy	125.52 kJ (30 kcal)/kg		
Insufficient energy	Amino Acids (17) and Glucose (1%) Injection (1440 mL/bag)		

SDFEN: soluble dietary fiber enteral nutrition. EN: enteral nutrition. Dietary fiber: Soluble dietary fiber from fruit and vegetables made by Beijing yikang Biotechnology Corp. (Beijing, China); Enteral Nutrition: Enteral Nutritional Emulsion (TP-HE) (500 mL/bag, 750 kcal) made by Sino-Swed Pharmaceutical Corp; Probiotics: Live Combined Bifidobacterium and Lactobacillus Tablets made by Inner Mongolia shuangqi Pharmaceutical Corp. (Huhhot, China).

**Table 2:** Characteristics of the cases and control.

Variable	SDFEN (*n* = 30)	EN (*n* = 30)	*p*
Gender (%)			
Male	16 (53.3)	19 (63.3)	0.735
Female	14 (46.7)	11 (36.7)	0.690
Age mean (SD) year	52.2 ± 14.2	53.2 ± 13.1	0.750
Smoking status (%)			
Smokers	12 (40)	9 (30)	0.664
Nonsmokers	18 (60)	21 (70)	0.749
Drinking (%)			
Drinkers	10 (33)	13 (43.3)	0.678
Nondrinkers	20 (67)	17 (56.7)	0.868
TNM stage (%)			
I, II	13 (43.3)	11 (36.7)	0.839
III	17 (56.7)	19 (63.3)	0.868

SDFEN: soluble dietary fiber enteral nutrition; EN: enteral nutrition; SD: standard deviation.

**Table 3:** Comparison of the two groups’ nutrition index.

	Pre Op	POD 2	POD 7
ALB (g/L)			
SDFEN	40.15 ± 2.45	32.64 ± 2.32	35.45 ± 2.21
EN	39.39 ± 2.87	31.81 ± 1.72	34.30 ± 1.86
*p*	0.22	0.09	0.05
PAB (mg/L)			
SDFEN	194.78 ± 31.74	177.32 ± 22.82	192.67 ± 21.30
EN	191.47 ± 30.61	178.09 ± 28.24	189.97 ± 25.32
*p*	0.62	0.88	0.58
TRF (g/L)			
SDFEN	2.58 ± 0.50	2.05 ± 0.39	2.29 ± 0.33
EN	2.55 ± 0.39	2.09 ± 0.20	2.23 ± 0.28
*p*	0.75	0.57	0.30

Pre Op: pre-operation; POD: postoperative day; SDFEN: soluble dietary fiber enteral nutrition; EN: enteral nutrition; ALB; albumin; PAB; prealbumin; TRF; transferring.

**Table 4:** Comparison of the two groups’ immune index.

	Pre Op	POD 2	POD 7
CD3+ (%)			
SDFEN	62.77 ± 6.46	54.32 ± 6.95	61.34 ± 4.32
EN	60.99 ± 7.00	53.59 ± 6.42	59.80 ± 6.23
*p*	0.321	0.669	0.285
CD4+ (%)			
SDFEN	34.28 ± 4.83	28.40 ± 3.32	35.90 ± 2.24
EN	33.70 ± 5.31	29.76 ± 3.32	34.41 ± 2.64
*p*	0.61	0.155	0.024
CD8+ (%)			
SDFEN	23.20 ± 4.83	21.58 ± 3.91	21.30 ± 3.03
EN	22.03 ± 3.83	21.11 ± 2.72	21.35 ± 1.94
*p*	0.377	0.659	0.949
CD4+/CD8+			
SDFEN	1.55 ±0.43	1.36 ± 0.31	1.72 ± 0.29
EN	1.58 ±0.41	1.44 ± 0.27	1.62 ± 0.20
*p*	0.766	0.387	0.190
IgA (g/L)			
SDFEN	2.13 ± 0.95	1.81 ± 0.94	2.25 ± 0.79
EN	2.03 ± 0.56	1.69 ± 0.59	1.88 ± 0.54
*p*	0.622	0.567	0.045
IgM (g/L)			
SDFEN	1.26 ± 0.64	0.94 ± 0.20	1.30 ± 0.33
EN	1.12 ± 0.19	0.96 ± 0.13	1.11 ± 0.15
*p*	0.241	0.693	0.002
IgG (g/L)			
SDFEN	11.22 ± 1.54	9.57 ± 1.59	11.55 ± 1.44
EN	11.12 ± 1.73	9.74 ± 0.87	11.41 ± 1.32
*p*	0.799	0.618	0.590

Pre Op: pre-operation; POD: postoperative day; SDFEN: soluble dietary fiber enteral nutrition; EN: enteral nutrition.

**Table 5:** Comparison of the two groups’ immune index.

	Pre Op	POD 2	POD 7
TNF-α (pg/mL)			
SDFEN	12.88 ± 5.62	20.32 ± 6.35	13.02 ± 2.85
EN	13.91 ± 6.47	22.14 ± 6.81	14.73 ± 4.07
*p*	0.524	0.333	0.045
IL-6(pg/mL)			
SDFEN	47.54 ± 34.30	110.72 ± 46.86	58.75 ± 24.82
EN	45.83 ± 42.14	115.57 ± 56.93	70.83 ± 35.65
*p*	0.850	0.682	0.044
PCT (ng/mL)			
SDFEN	0.11 ± 0.11	0.26 ± 0.18	0.15 ± 0.07
EN	0.15 ± 0.15	0.28 ± 0.18	0.16 ± 0.08
*p*	0.086	0.489	0.539

Pre Op: pre-operation; POD: postoperative day; SDFEN: soluble dietary fiber enteral nutrition; EN: enteral nutrition.

**Table 6:** Post-operative recovery.

	SDFEN	EN	*p*
First flatus time (h)	58.93 ± 6.5	63.03 ± 4.8	0.015
Abdominal distension	1	2	-
Pneumonia	1	2	-
Anastomotic fistula	0	1	-
Length of hospital stay (day)	7.4 ± 0.72	7.63 ± 0.61	0.257

SDFEN: soluble dietary fiber enteral nutrition; EN: enteral nutrition.
